# Supplementary material for: Single‐Sequence Deep Learning Delivers Crystal‐Quality Models of Covalent K‐Ras G12 Hotspot Complexes
Source: IUBMB Life. 2026 Jun 13;78(6):e70108. doi: 10.1002/iub.70108 (PMC13263772; doi:10.1002/iub.70108)
Supplement: Supplementary file 2 — Table S1: Experimental structures used as ground‐truth references for pose comparison. Unless otherwise indicated, structures correspond to K‐Ras(G12C) in the GDP‐bound state. 1G12Di‐1 corresponds to K‐Ras(G12D). 2G12Si‐5 corresponds to K‐Ras(G12S). For BBO‐8520, PDB 8V3A corresponds to the GDP‐bound state and PDB 8V39 corresponds to the GMPPNP‐bound active‐state structure. Table S2: Geometric pocket‐occupancy metrics for Chai‐1 Divarasib predictions in WT K‐Ras and resistance‐associated K‐Ras(G12C) variants without covalent restraint. WT K‐Ras‐Divarasib‐GDP‐Mg2+ and K‐Ras(G12C) secondary Switch‐II pocket variant predictions were performed in Chai‐1 single‐sequence mode without covalent restraints. Pocket distance indicates the ligand centroid distance to the centroid of the pocket‐defining residues 12, 68, 72, 95, 96, and 99. Pocket contacts indicate the number of ligand atoms within 4.0 Å of any atom in these pocket‐defining residues. Rank 0 values describe the top‐ranked prediction, and cross‐rank values summarize pose variability across ranks 0 to 4. [file IUB-78-0-s002.docx]

**Supplementary TablES**

| **Ligand** | **PDB ID** |
| --- | --- |
| ARS-853 | 5F2E |
| ARS-1620 | 5V9U |
| Sotorasib / AMG 510 | 6OIM |
| Adagrasib / MRTX849 | 6UT0 |
| Divarasib / GDC-6036 | 9DMM |
| JDQ443 | 7R0M |
| BBO-8520 | 8V3A, 8V39 |
| G12Di-1^1^ | 8T4V |
| G12Si-5^2^ | 7TLG |
| Cpd3 | 7A1W |
| Cpd4 | 7A47 |

**Supplementary Table S1 | *Experimental structures used as ground-truth references for pose comparison.*** Unless otherwise indicated, structures correspond to K-Ras(G12C) in the GDP-bound state. ^1^G12Di-1 corresponds to K-Ras(G12D). ^2^G12Si-5 corresponds to K-Ras(G12S). For BBO-8520, PDB 8V3A corresponds to the GDP-bound state and PDB 8V39 corresponds to the GMPPNP-bound active-state structure.

| **Variant** | **Rank 0 pocket distance (Å)** | **Rank 0 pocket contacts** | **Max ligand RMSD across ranks 0-4 (Å)** | **Minimum pocket contacts across ranks 0-4** |
| --- | --- | --- | --- | --- |
| WT K-Ras | 5.22 | 8 | 1.46 | 7 |
| K-Ras(G12C) | 4.12 | 26 | 1.70 | 25 |
| K-Ras(G12C/H95D) | 3.93 | 33 | 2.96 | 30 |
| K-Ras(G12C/H95L) | 4.45 | 33 | 1.71 | 26 |
| K-Ras(G12C/H95Q) | 3.87 | 29 | 6.49 | 29 |
| K-Ras(G12C/H95R) | 4.44 | 31 | 3.42 | 31 |
| K-Ras(G12C/M72I) | 4.01 | 33 | 1.24 | 31 |
| K-Ras(G12C/Q99L) | 4.18 | 27 | 2.45 | 25 |
| K-Ras(G12C/R68M) | 4.44 | 30 | 2.00 | 28 |
| K-Ras(G12C/R68S) | 4.11 | 27 | 6.42 | 25 |
| K-Ras(G12C/Y96C) | 4.20 | 23 | 7.02 | 22 |
| K-Ras(G12C/Y96D) | 4.22 | 25 | 1.37 | 20 |
| K-Ras(G12C/Y96H) | 4.46 | 28 | 1.37 | 28 |
| K-Ras(G12C/Y96N) | 4.44 | 21 | 2.80 | 17 |
| K-Ras(G12C/Y96S) | 4.45 | 22 | 1.35 | 21 |

**Supplementary Table S2 | *Geometric pocket-occupancy metrics for Chai-1 Divarasib predictions in WT K-Ras and resistance-associated K-Ras(G12C) variants without covalent restraint.*** WT K-Ras-Divarasib-GDP-Mg^2+^ and K-Ras(G12C) secondary Switch-II pocket variant predictions were performed in Chai-1 single-sequence mode without covalent restraints. Pocket distance indicates the ligand centroid distance to the centroid of the pocket-defining residues 12, 68, 72, 95, 96, and 99. Pocket contacts indicate the number of ligand atoms within 4.0 Å of any atom in these pocket-defining residues. Rank 0 values describe the top-ranked prediction, and cross-rank values summarize pose variability across ranks 0 to 4.
